# Mechanisms of Ethanol-Induced Cerebellar Ataxia: Underpinnings of Neuronal Death in the Cerebellum

**DOI:** 10.3390/ijerph18168678

**Published:** 2021-08-18

**Authors:** Hiroshi Mitoma, Mario Manto, Aasef G. Shaikh

**Affiliations:** 1 Medical Education Promotion Center, Tokyo Medical University, Tokyo 160-0023, Japan; 2 Unité des Ataxies Cérébelleuses, Service de Neurologie, CHU-Charleroi, 6000 Charleroi, Belgium; mmanto@ulb.ac.be; 3 Service des Neurosciences, University of Mons, 7000 Mons, Belgium; 4 Louis Stokes Cleveland VA Medical Center, University Hospitals Cleveland Medical Center, Cleveland, OH 44022, USA; axs848@case.edu

**Keywords:** alcohol, ethanol, cerebellum, cerebellar ataxias, fetal alcohol spectrum disorder

## Abstract

Ethanol consumption remains a major concern at a world scale in terms of transient or irreversible neurological consequences, with motor, cognitive, or social consequences. Cerebellum is particularly vulnerable to ethanol, both during development and at the adult stage. In adults, chronic alcoholism elicits, in particular, cerebellar vermis atrophy, the anterior lobe of the cerebellum being highly vulnerable. Alcohol-dependent patients develop gait ataxia and lower limb postural tremor. Prenatal exposure to ethanol causes fetal alcohol spectrum disorder (FASD), characterized by permanent congenital disabilities in both motor and cognitive domains, including deficits in general intelligence, attention, executive function, language, memory, visual perception, and communication/social skills. Children with FASD show volume deficits in the anterior lobules related to sensorimotor functions (Lobules I, II, IV, V, and VI), and lobules related to cognitive functions (Crus II and Lobule VIIB). Various mechanisms underlie ethanol-induced cell death, with oxidative stress and endoplasmic reticulum (ER) stress being the main pro-apoptotic mechanisms in alcohol abuse and FASD. Oxidative and ER stresses are induced by thiamine deficiency, especially in alcohol abuse, and are exacerbated by neuroinflammation, particularly in fetal ethanol exposure. Furthermore, exposure to ethanol during the prenatal period interferes with neurotransmission, neurotrophic factors and retinoic acid-mediated signaling, and reduces the number of microglia, which diminishes expected cerebellar development. We highlight the spectrum of cerebellar damage induced by ethanol, emphasizing physiological-based clinical profiles and biological mechanisms leading to cell death and disorganized development.

## 1. Introduction

Chronic alcohol consumption is associated with social and medical consequences. A major public health problem in many countries, the prevalence of chronic alcoholism is 0.5–3% in Europe and the USA [1]. Ethanol ingestion affects a number of systems, including the central nervous system (CNS) and the peripheral nervous system (PNS). The mature or developing cerebellum is one of the most vulnerable areas affected by alcohol consumption. The effects of alcohol on the nervous system are either acute and transient or chronic and permanent. They are summarized in Table 1.

Cerebellum is one of the most susceptible targets. The acute and transient effects of alcohol are characterized by impaired posture, ataxic gait, and scanning speech collectively termed alcoholic cerebellar ataxia [2] or ethanol-induced cerebellar ataxia [3]. Most patients complain of a lack of coordination in the lower extremities, contrasting with the relative sparing of upper limbs, suggesting a preferential dysfunction of the vermis. Chronic alcoholism is associated with cerebellar atrophy, especially the anterior superior vermis above the primary fissure. Two mechanisms have been depicted that potentially underly this etiology [4]. One possibility underscored the role of nutritional deficits as a cause of cerebellar atrophy, hence the term “nutritional cerebellar degeneration” [2,5,6]. The second mechanism emphasized the direct effects of ethanol on neurons and glia through multiple pathways that converge on oxidative stress and endoplasmic reticulum (ER) stress on the cellular components of the cortex [7]. In addition, the developing cerebellum is highly vulnerable to the toxic effects of ethanol [7]. Children with fetal alcohol spectrum disorder (FASD) show various symptoms, including deficits in general intelligence, attention, executive function, language, learning, visual perception, and social skills [8,9]. Some of these symptoms can be attributed to differentiation deficits in the cerebellar cortex and subsequent impairment in cerebellar coordinative controls on cognitive functions through the cerebello–cerebral loops.

Ethanol is metabolized to acetaldehyde by alcohol dehydrogenase and cytochrome p450 2E1 and subsequently to acetic acid by aldehyde dehydrogenases [10]. Intracellular generation of free radicals and depletion of the antioxidant glutathione are vital steps in the cellular pathogenic events caused by ethanol [10]. In addition to such a classic view, recent studies using various animal models have shown diverse pathophysiological mechanisms. For example, Zebrafish models clarified that ethanol exposure impaired mitochondrial bioenergetics, leading to a production of reactive oxygen species [11]. Furthermore, since ethanol metabolism is multifaceted, mouse models with genetic deficiencies in ethanol-metabolizing enzymes led to a deeper understanding of the multi-layered molecular pathways [10]. Similarly, studies using various methodologies have facilitated the elucidation of the mechanisms by which ethanol exposure impairs the adult and fetal cerebellum. Interestingly, ethanol interferes with the differentiation in the fetal cerebellum through oxidative and ER stresses, as found in the adult cerebellum [12].

Here, we will summarize the clinical profiles of cerebellar ataxias (CAs) that are observed after alcohol consumption and in those with FASD. We will discuss recent progress in physiological and molecular biological mechanisms underlying cerebellar ataxia. We focus on the mechanisms leading to neuronal death. The review algorithm is shown in Supplement to show our search for this review.

## 2. Acute Effects of Alcohol

### 2.1. Clinical Profiles

Acute alcohol intoxication is a harmful condition that follows the ingestion of a large amount of alcohol [13]. Ethanol, at low concentrations (>0.08 g/L), interferes with cerebellar functions [3]. Ataxia appears immediately after acute alcohol consumption. The deficits include gait instability, anterior–posterior oscillations in Romberg’s test, and various degrees of dysarthria. Veering and staggering are typical and are not associated with a trigger of compensatory action such as widening of stance [2], the symptom contrasting with those in chronic alcoholism (see below Section 3).

### 2.2. Pathophysiology

Synaptic dysfunction in the cerebellar cortex is at the hallmark of ataxia after acute alcohol intoxication. Two synaptic locations are particularly vulnerable to the acute effects of alcohol: the synapses on the granule cells (GCs) that gate the cerebellar cortex and those on the Purkinje cells (PCs), which represent the cerebellar cortical output [3].

#### 2.2.1. Deafferentation

**Figure 1:**
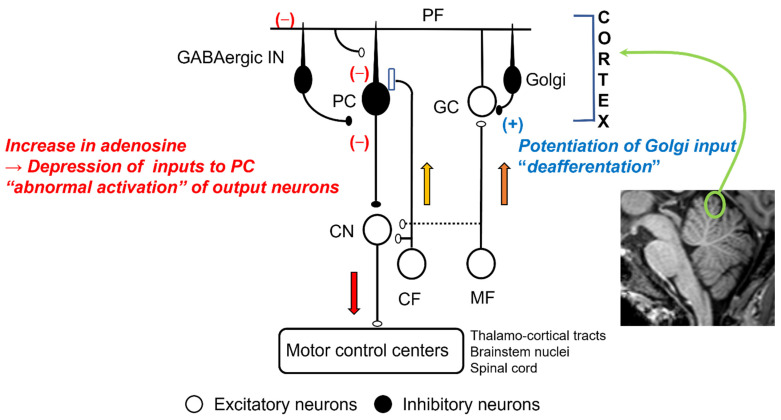
Effects of ethanol in cerebellar synaptic transmissions: MF, mossy fiber; CF, climbing fiber; GC, granule cell; Golgi, Golgi cell; PF, parallel fiber; GABAergic In, GABAergic interneurons; CN, cerebellar nucleus neurons.

Mossy fibers conveying the afferent information from the periphery and the cerebral cortex terminate on the GCs [14]. The activities of GCs are under feedback control from the Golgi cells [14] (Figure 1). Ethanol directly enhances the inhibitory synaptic transmission between Golgi cells and GCs via two mechanisms. First, ethanol inhibits NA^+^/K^+^ATPase, a quinidine-sensitive K^+^ channel, and neuronal nitric oxide (NO) synthesis, which causes activation of Golgi cells [15]. Second, ethanol potentiates extrasynaptic GABA_A_ receptors on GCs [7]. These pre- and post-synaptic effects of ethanol facilitate GABA_A_-mediated synaptic transmission [16], which results in an attenuation of the inputs essential for the cerebellar control, i.e., the cerebellar “deafferentation” [3] (Figure 1).

#### 2.2.2. Abnormal Activation of Outputs Cells

**Figure 2:**
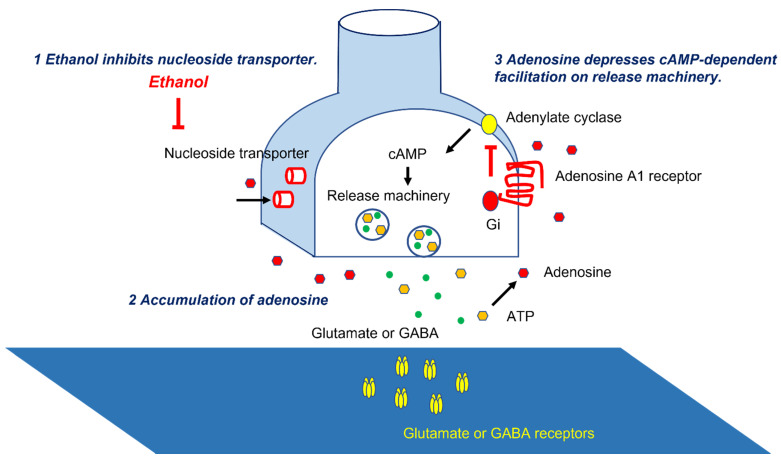
Molecular mechanisms underlying adenosine-induced synaptic depression. Ethanol inhibits nucleoside transporters, leading to accumulation of adenosine. Adenosine depresses cAMP-dependent facilitation on release machineries.

The parallel fibers (PFs), the long axons of the GCs, activate arrays of PCs [14]. PFs also activate inhibitory interneurons (Basket cells and stellate cells), suppressing PCs [14]. Thus, PCs’ activities are under the control of both excitatory and inhibitory inputs (Figure 1). These inputs on PCs are modulated by adenosine. Adenosine, present in the soma and dendrites of PCs [17], inhibits the release of glutamate and GABA from the synaptic terminals through a decrease in cAMP [3,18,19,20,21]. The source of synaptic adenosine is the breakdown of metabolites of ATP released from PFs [22]. Furthermore, nucleoside transporters also regulate extraneuronal and synaptic levels of adenosine [23]. Ethanol blocks the uptake of adenosine through the inhibition of ethanol-specific nucleoside transporter (ENT1) [24,25] (Figure 2). Subsequently, there is an accumulation of adenosine in the cerebellar cortex and subsequent potentiation of adenosine-induced inhibition on various synapses, such as excitatory synapses between PFs and PCs and between PFs and Basket cells, and inhibitory synapses between Basket cells and PCs [3]. The net effect is inadequate activation of the PCs and, in turn, the output signals, i.e., “abnormal activation” (Figure 1) [3]. These abnormal patterns of discharges will have widespread effects on cerebellar targets.

Ethanol also inhibits the late phase of the climbing fiber-induced complex spike [26]. Since this is the result of the inhibition of metabotropic glutamate receptor type 1 (mGluR1) on post-synaptic PCs, ethanol also impairs the induction of long-term depression on PF–PC synapses, a form of critical synaptic plasticity dependent on mGluR1 [26].

In summary, deafferentation and abnormal activation by depressing the cerebellar inputs and the outputs disrupts motor and cognitive coordination.

## 3. Chronic Effects of Alcohol

### 3.1. Clinical Profile

Alcoholic cerebellar degeneration is a disorder of chronic alcohol dependence [27]. The disease generally evolves over weeks or months, but it may occur abruptly [27]. On the other hand, a study shows that daily consumption of 150 g of alcohol for 10 years was associated with significant cerebellar atrophy on CT in 30% of patients [28]. Gait and posture instability is a characteristic cerebellar symptom of chronic alcoholism. Peculiar gait abnormalities include a massive sway and irregular stepping, associated with a compensatory short stride, widened stance, and slow speed [2,29]. Lower limb ataxia is common and obvious during heel–shin testing. More advanced cases also have upper limb ataxia and dysarthria. A 3-Hz postural leg tremor is very suggestive. Legs may appear stiff during gait but not in the lying position. Gait difficulties either worsen progressively over weeks or months or rapidly turn to a debilitating stage in the case of malnutrition [1]. A third pattern of progression is an unpredictable exacerbation. Neuropsychological studies have shown impairments in cognitive functions, especially executive skills in the Category Test [30]. However, since alcoholic patients without cerebellar degenerations show similar cognitive deficits, severe cerebellar cognitive dysfunctions are not likely to occur [1].

Vermis atrophy is the most common feature of chronic alcoholism [2]. The sagittal plane on T1-weighted MRI images shows a clear atrophied area, which is often easy to identify and demarcate. Positron emission tomography (PET) shows hypoperfusion of the atrophic region [2]. It is estimated that between 27% and 42% of patients presenting with alcoholism have cerebellar degeneration [31,32,33]. On the other hand, it is not exceptional to observe severe cerebellar atrophy on imaging in younger chronic alcohol-dependent patients without cerebellar signs. However, cerebellar symptoms become manifest after follow-up [1].

### 3.2. Pathophysiology

#### 3.2.1. Pathology

Chronic alcoholism is characterized by the atrophy of the anterior superior vermis (anterior lobe), with a reduction in white matter; the severity of structural deficits correlates with that of CA [2,29]. The microscopic findings include narrowing of the molecular layer, loss of PCs, and patchy loss of granule cells [29,34]. The reported incidence of typical histopathological changes (atrophy of the anterior superior vermis) varies from 0.4% (in 2664 autopsies) [35] to 4.0% (in 3578 autopsies) [36].

#### 3.2.2. Malnutrition-Induced Degeneration

A characteristic distribution of vermis atrophy is found in patients with alcoholic and nonalcoholic Wernicke’s encephalopathy [37,38,39]. Nonalcoholic Wernicke’s encephalopathy is caused by three etiologies: absolutely low intake (staple diet of polished rice, unbalanced nutrition, psychogenic food refusal, recurrent vomiting, diarrhea, hyperemesis gravidarum, thyrotoxicosis, prolonged parenteral nutrition without vitamin supplementation, and intestinal surgery), malabsorption due to surgical resection, and relatively low intake in cases with increased demand (cancer, chemotherapy, and systemic disease) [40]. There are no consistent changes in the number of neurons or structural volume loss in chronic alcoholics without Wernicke’s encephalopathy; however, cell loss was found in thiamine-deficient chronic alcoholics (43% reduction in PCs and 32% reduction in molecular layer volume) [41]. These results suggest that PCs are selectively vulnerable to thiamine deficiency and that generalized cerebellar atrophy could be due to global malnutrition due to thiamine (vitamin B1) deficiency [2]. Other factors such as deficiency of folate, pyridoxine, and zinc might amplify the malnutrition-induced deficiency [42].

Thiamine, a water-soluble essential vitamin, is stored in the liver as thiamine diphosphate (TDP), a cofactor for critical enzymes of the Krebs cycle (TCA cycle) and pentose phosphate cycle (pyruvate dehydrogenase complex, α-ketoglutarate dehydrogenase complex, and transketolase). Through the Krebs cycle and pentose phosphate cycle, thiamine is involved in energy production by ATP synthesis, lipid metabolism (production of myelin sheath), and production of amino acids and glucose-derived neurotransmitters (glutamate and GABA) [40,43,44]. Thus, a decrease in thiamine elicits diverse changes that can lead to cell death [40,43,44]. First, entry of pyruvate to the Krebs cycle is debilitated, which results in a deficiency in cellular energy. Second, a decrease in thiamine impairs the pentose phosphate cycle, which leads to an increase in extracellular glutamate. Accumulation of α-ketoglutarate increases glutamate synthesis through transamination, resulting in a subsequent release of glutamate into the extracellular space. Simultaneously, a decrease in α-ketoglutarate dehydrogenase worsens astrocytes, causing a reduction in glutamate uptake. The excessive glutamate increases Ca^2+^ influx, which causes excitotoxicity, characterized by the subsequent association of compound pro-apoptosis pathways, such as mitochondrial dysfunction, oxidative stress, endoplasmic reticulum (ER) stress, and DNA damage [45]. Third, thiamine-deficient membranes are unable to maintain osmotic gradients, which causes intracellular edema with swelling of neuronal dendrites, oligodendrocytes, and myelin sheets [1]. Fourth, thiamine deficiency characteristically insults particularly vulnerable regions: the periventricular and periaqueductal regions, where thiamine-related glucose and oxidation metabolism are abundant with high thiamine content and turnover [40]. Finally, electrolyte disorders are also implicated in thiamine-induced degeneration [46].

A personal susceptibility is well-known in chronic alcoholic toxicity [47]. Cerebellar degeneration is not correlated with the dose ingested [48]. One study reported an ataxic patient with a history of small doses of alcohol over 15 years. Interestingly, the intake of small quantities of alcohol (5 g) prominently exaggerated his ataxia (e.g., induction of gaze-evoked nystagmus, a scanning speech, a body sway after eye closure, and bilateral postural leg tremors) [47]. Different levels of affinity between thiamine pyrophosphatase and transketolase were reported [49], suggesting personal vulnerability might be attributed to individual differences in the thiamine enzyme system [1].

In addition to thiamine deficiency, associated liver dysfunction can indirectly exaggerate the toxic effects via an imbalance in amino acids and the production of neurotoxic metabolic and inflammatory factors, leading to cerebellar degeneration [50,51].

#### 3.2.3. Ethanol-Induced Direct Neuronal Degeneration

Recent animal experiments have demonstrated that ethanol can directly cause cerebellar degeneration and that such an effect is potentiated by aging [52]. Chronic exposure to alcohol induces oxidative stress through a multitude of sources. For example, ethanol and its metabolites damage the mitochondria, which leak reactive oxygen species (ROS). Furthermore, ethanol activates NADPH oxidase and induces oxidative/peroxidative/epoxidation from membrane polyunsaturated fatty acids (especially arachidonic acid) [53]. Oxidative stress is not necessarily downstream of excitotoxicity [53]. On the other hand, chronic ethanol consumption elicited regression of PC dendritic arbors, preceded by swelling of the smooth endoplasmic reticulum (SER) in aging rats [52]. Ethanol also interfered with the sarco/endoplasmic reticulum Ca^2+^ ATPase pump (SERCA), which is responsible for the re-sequestration of Ca^2+^ into the SER, leading to ER stress [52].

#### 3.2.4. Genetic Expression Factors

It has been a focus of interest how gene expression is altered during alcohol abuse [54]. Ethanol exposure alters chromatin architecture via DNA methylation and histone acetylation, which ultimately affects gene expression and behavior [54]. Fragile-X mental retardation protein (FMRP) is a complex regulator of RNA that represses translation by stalling bound targets and stabilizing synaptic mRNA transcripts [54]. Fragile-X tremor and ataxia syndrome (FXTAS), a Fragile-X mental retardation (*FMR1*) gene-related-phenotype, results from a pre-mutation condition with 55–200 200 CGG trinucleotide repeats and is characterized by paradoxically increased mRNA expression of *FMR1* [55]. Alcohol abuse causes earlier onset and greater severity of FXTAS. Thus, it is proposed that CAs are partially mediated by epigenetic regulation of *FMR1* expression in the cerebellum [54]. Similar pathologies can occur in hereditary degenerative CA [56].

#### 3.2.5. Long-Term Alcohol Exposure

Long-term alcohol exposure affects receptors and transporters in several neurotransmitter systems, including glutamate, GABA, glycine, norepinephrine, serotonin, dopamine, and acetylcholine [57]. A study using ^11^C-flumazenil showed that chronic alcoholism reduced neurons containing GABA_A_/benzodiazepine receptors in the superior cerebellar vermis [58]. Glucose hypometabolism in the anterior and superior portions of cerebellar vermis was also found in chronic alcoholic ataxic patients [27,59]. In addition, chronic alcohol inhibits NMDA receptors, and prolonged alcohol exposition causes upregulation of NMDA receptors [57].

Alcohol withdrawal syndrome occurs in alcohol-dependent patients after a period of excessive use, which includes anxiety, shakiness, and sweating [60,61]. The withdrawal-induced release of glutamate in the cerebellar cortex [61] elicits excitotoxicity of PCs and GCs.

**Figure 3:**
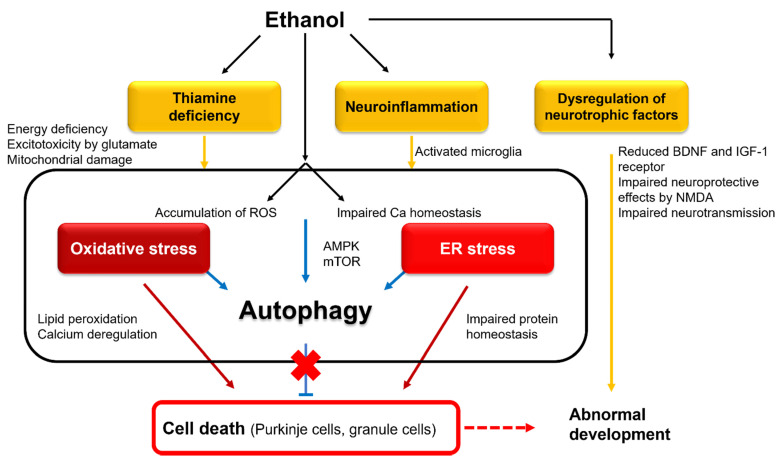
Intracellular mechanisms underlying ethanol-induced cell death and abnormal development: BDNF, brain-derived neurotrophic factor; NMDA, N-methyl-D-aspartate; ROS, reactive oxygen species; AMPK, AMP-activated protein kinase; mTOR, mammalian target of rapamycin.

In conclusion, both malnutrition and the direct ethanol effect contribute to cerebellar atrophy in chronic alcohol use. However, it is noteworthy that multiple causes reflect the trigger of cell death, and these mechanisms converge to form a final common pathway leading to the activation of pro-apoptotic cascades, such as oxidative stress, ER stress, and DNA damage (Figure 3).

### 3.3. Therapies and Prognosis

Abstinence is the recommended therapy for alcohol-dependent patients. Victor et al. [6] proposed thiamine replacement therapy at a recommended daily dose of 100 mg. The effectiveness of such therapy in at least some patients provides strong evidence for the pathogenic role of malnutrition in CAs. Long-term follow-up is necessary to lessen the chance of recidivism [60]. One long-term follow-up study showed that successful abstinence was associated with stabilization of the severity of CAs after decreasing from the peak observed in days to months after abstinence in 50% of patients, whereas 35% of patients continued the abuse and had progressive CAs [29]. In 15% of patients, CA was almost stable over a few years, though it subsequently showed abrupt deterioration.

Rehabilitation and clinical and neuropsychological follow-up are recommended. It is likely that social and cognitive rehabilitation will emerge in the coming years, given the key roles of the cerebellum in social interactions/cognitive operations and the social consequences of chronic ethanol consumption [62]. For the treatment of alcohol use disorders (AUD), only a few medications are available, but they have modest effects. They include naltrexone, an opioid antagonist, and acamprosate, a putative glutamate receptor antagonist [63,64]. Supervised administration of disulfiram, an aldehyde dehydrogenase inhibitor, is a second-line therapy [63,64]. Encouraging results have been obtained for other medications, including topiramate, gabapentin, ondansetron, varenicline, baclofen, sodium oxybate, and antidepressants, but further studies are needed [63].

Thiamine should be administered by the intravenous route without delay in Wernicke’s encephalopathy. Electrolyte disorders should be corrected. Techniques of non-invasive stimulation of the cerebellum are promising on the basis of the high reactivity of cerebellar circuitry to neuromodulation [65].

## 4. Fetal Alcohol Spectrum Disorders

Alcohol has teratogenic effects on prenatal development [42]. Fetal alcohol spectrum disorders (FASD) are defined as a range of permanent congenital disabilities caused by maternal alcohol consumption during pregnancy [66]. Imaging and autopsy studies of the affected individuals showed reductions and abnormalities in overall brain size and shape, especially in the cerebellum, basal ganglia, and corpus callosum [67].

### 4.1. Clinical Profiles

Prenatal exposure to ethanol has a negative impact on various cognitive domains [68]. Decreased intelligence quotient (IQ) is one of the most typical findings in FASD. The reported average IQ of children with FASD ranges from 68 to 79 [69]. Attention-deficit is also a common symptom in children with FASD. The overall performance in affected children is slower due to difficulties in sustaining attention [68]. Furthermore, the processing of visually presented information in children with FASD was associated with a high number of omission errors [70]. Other significant impairments are observed in the executive function, including verbal fluency, inhibition, problem-solving and planning, concept formation, set-shifting and working memory, language abilities of articulation, grammatical skills, and expressive/receptive skills, memory such as encoding and recall, visual perception and construction tasks, communication, and socialization [68]. Children with FASD also have impairments of motor skills, including deficits in fine motor control and hand-eye coordination [71,72]. Although these symptoms cannot be attributed to dysfunction of a specific brain region, children with FASD develop the core symptoms that are consistent with cerebellar dysfunction, such as ataxic motor symptoms and cognitive symptoms (e.g., deficits in attention sustaining, verbal fluency, visual perception, and social skills) [73,74]. It is likely that the disorganization of cerebellar controls in cognition is one of the critical factors in FASD. Consistent with this notion, a recent MRI study of children with FASD showed volume deficits in the anterior lobules related to sensorimotor functions (Lobules I, II, IV, V, and VI) and lobules related to cognitive functions (Crus II and Lobule VIIB) [75].

### 4.2. Pathophysiology

Rodent models of fetal ethanol exposure have shown that the PCs and GCs are vulnerable to damage with ethanol in the developing cerebellum [76,77,78]. Blood alcohol concentrations of >180 mg/dL were associated with PCs depletion during postnatal days 4–9 (part of the third trimester of human gestation equivalent), a period corresponding with PC dendritic outgrowth and synaptogenesis [79]. Such depletion showed regional differences, with the most marked PCs depletion in Lobules I–V and VIII–X. Compared to PCs, GCs were more vulnerable to ethanol at the same blood concentration [78]. The toxic effects of ethanol were noted during postnatal day 8–9 [77,80], suggesting a temporal window of vulnerability to ethanol exposure during fetal development [66].

#### 4.2.1. Interference with Neurotrophic and Retinoic Acid Pathways

Cerebellar development is controlled by various neurotrophic factors, including insulin and insulin-like growth factor (IGF-1) [81] and brain-derived neurotrophic factor (BDNF) [82]. Exposure to ethanol during fetal life reduced the expression of IGF-1 receptors [83] and downregulated BDNF mRNA [82] in GCs, thereby inhibiting these neurotrophic factor-mediated signals. These neurotrophic factors are also driven by N-methyl-D-aspartate (NMDA) in cerebellar immature GCs [84]. Thus, it seems that NMDA effectively facilitates normal differentiation of GCs, which undergo innervation by glutamatergic synapses [66]. Ethanol also impairs the neuroprotective effects of NMDA [85]. Ethanol inhibits neural activities through the inhibition of NMDA receptors and, furthermore, the facilitation of GABA_A_ receptors, leading to massive neuroapoptosis in the developing brain [86,87,88]. Taken together, interference with these neurotrophic and neuroprotective factors is assumed to explain abnormal fetal neural development [66] (Figure 3).

Retinoic acid (RA) is synthesized in the cerebellum and acts as an endogenous regulator of cerebellar development [89]. Ethanol increased the expression of rexinoid (retinoid X) receptors, one of the RA receptors in GCs, in the cerebellum at postnatal day 7 [90]. Since rexinoid and its target gene are predominantly involved in apoptosis, ethanol might also exert its harmful effects through RA receptor-mediated signals [66].

#### 4.2.2. Involvement of Oxidative and ER Stresses

Exposure to ethanol at postnatal day 4 increased intracellular ROS [91] and simultaneously decreased the activity of antioxidative enzymes in GCs [92]. In this regard, the accumulation of intracellular ROS induces oxidative stress, with the destruction of the mitochondria membranes and leakage of cytochrome c, resulting in caspase activation and cell apoptosis [66] (Figure 3). On the other hand, ethanol induced an unfolded protein response (UPR) [93]. UPR triggered a response in ER stress in the whole brain of postnatal day 7 mice, indicated by the upregulation of ER stress sensors, such as pancreatic endoplasmic reticulum kinase (PERK), inositol-requiring enzyme 1 (IRE1), and activating transcription factor 6 (ATF6) [93]. These findings suggest that ethanol-induced ER stress can trigger neuroapoptosis in the cerebellum (Figure 3). Although mechanisms underlying ER stress are unknown, various disturbances such as the disruption of intracellular calcium homeostasis or redox status are implicated [93].

#### 4.2.3. Autophagic Protection

Recent studies have shown that ethanol promotes autophagic flux in neurons [12]. Defective mitochondria were eliminated by selective autophagy, which protects against mitochondrial release of pro-apoptotic factors and ROS accumulation [12]. However, prolonged exposure to ethanol impaired the autophagy–lysosome pathway [94]. Thus, prenatal exposure to ethanol seems to affect autophagic protection against oxidative and ER stresses, which results in the acceleration of fetal developmental deficits (Figure 3).

#### 4.2.4. Neuroinflammation

Microglia has two aspects of functions. Microglia protects the CNS by removing potentially neurotoxic substances and is involved in the formation of functional synapses by synaptic pruning. In contrast, after an insult to the CNS, microglia are activated to produce various pro-inflammatory molecules, such as cytokines, ROS, and NO, with subsequent neurodegeneration [95,96]. Microglia activation is mediated by Toll-like receptors (TLRs) with conserved motifs associated with the pathogens [96].

Ethanol decreases the number of microglia, reduces their ability to protect neurons, and dampens their role in normal neuronal development [96]. The behavioral consequences of microglial depletion were relevant to FASD [96]. On the other hand, the surviving microglia were activated [96], and such activation was blocked by agonists of the anti-inflammatory nuclear receptor PPAR-γ to protect against ethanol-induced neuronal loss [96]. TNF-α neutralizing antibodies blocked ethanol-induced neuronal death [97]. These studies suggest that ethanol activates microglia to secret pro-inflammatory cytokines, causing the loss of neurons in the developing brain (Figure 3).

In conclusion, ethanol impairs neurotrophic factors and retinoic acid pathways and decreases the number of microglia, both of which result in cerebellar developmental deficits. In addition, ethanol recruits directly or indirectly various pro-apoptotic pathways, including oxidative stress, ER stress, and neuroinflammation. The collapse of the autophagic protection machinery enhances cooperation among these divergent pro-apoptotic pathways to cause neuronal death.

### 4.3. Intervention

Multifaceted interventions are used in the absence of specific medicines for FASD [98]. Such interventions include parent training and support programs, parent–child interaction therapy, pay attention programs, cognitive control programs, and adaptive functioning programs. A recent randomized, double-blind, placebo-controlled study highlighted the therapeutic benefits of choline supplementation on attention, executive, and memory deficits in children with FASD [99]. It might be because choline facilitates neurogenesis, thereby contributing to increased cell proliferation and decreased apoptosis [100].

## 5. Conclusions: Crosstalk between Cell Degeneration and Abnormal Development

Alcohol consumption impacts noticeably on cerebellar circuitry. Acute alcohol intoxication causes ataxia and dysarthria. The impairment due to acute alcoholism is likely attributed to the lack of afferent connections to the cerebellum or malfunctioning of the cerebellar output, such as PCs. On the other hand, chronic alcohol consumption elicits vermis atrophy responsible for gait ataxia associated with variable degrees of lower limb ataxia. Alcohol consumption in pregnancy can have devastating effects on the fetus, i.e., FASD. The location of the affected region in FASD has been challenging to determine since experimental prenatal exposure to ethanol results in injury of a large area in the brain. However, there are similarities in symptoms between FASD and cerebellar cognitive-affective or Schmahmann syndrome, including deficits in attention sustaining, verbal fluency, visual perception, and social skills. Children with FASD show volume deficits in the lobules related to cognitive functions (Crus II and Lobule VIIB). These findings suggest that although FASD is attributed to overall brain damage, cerebellar dysfunction or cerebello–cerebrum loop impairment might be one of the possible lesions in the neuropsychiatric symptoms in FASD. Notably, such a dysfunction will be a combined manifestation of the operation of damaged cerebellar circuits and the maladaptive synaptic plasticity that operates for the compensation for lost function [101].

Adult and fetal cerebellar neurons are vulnerable to chronic ethanol exposure. In both pathologies, oxidative stress and ER stress are the core pro-apoptotic pathways [102]. These pathways are induced by a thiamine-deficient environment, especially in alcohol abuse, and exacerbated by neuroinflammation, especially fetal ethanol exposure. The interactions between ethanol, neuroinflammation, and the neuroimmune system open perspectives for therapies in FASD [103]. Although autophagy originally serves as a potential protective response to ethanol-induced neurotoxicity, prolonged exposure to ethanol dysregulates the autophagic protection against oxidative and ER stresses. Such overlapping pathological mechanisms suggest crosstalk between cell degeneration and abnormal development. Notably, these cascading mechanisms exist in other etiologies, such as ischemia and the growingly recognized immune-mediated cerebellar ataxias [104]. Thus, further studies on ethanol-induced cell death could provide new clues to the understanding of cerebellar cell death.

## Figures and Tables

**Table 1:** Neurological deficits associated with ethanol consumption.

**More Prevalent Neurological Deficits**
Ataxic stance, titubation
Ataxic gait
Peripheral neuropathy
Wernicke’s encephalopathy
Acute confusional state
Hallucinations
Agitation
Korsakoff syndrome
Alcohol withdrawal syndrome
Seizures, myoclonus, asterixis
**Less Prevalent Neurological Deficits**
Gaze-evoked nystagmus
Ocular dysmetria
Ophthalmoparesis
Ataxic speech
3 Hz postural leg tremor
Kinetic tremor
Hypotonia
Amyotrophy
Autonomic overactivity
Increased risk of stroke

## Data Availability

The concept reported in this manuscript is not associated with raw data.

## Supplementary Materials

The following are available online at https://www.mdpi.com/article/10.3390/ijerph18168678/s1, Figure S1: Review algorithm.

## Code Availability

There is no software application or custom code used in this paper.
