# Supplementary figures and images for: Mechanisms of Ethanol-Induced Cerebellar Ataxia: Underpinnings of Neuronal Death in the Cerebellum
Source: Int J Environ Res Public Health. 2021 Aug 18;18(16):8678. doi: 10.3390/ijerph18168678 (PMC8391842; doi:10.3390/ijerph18168678)

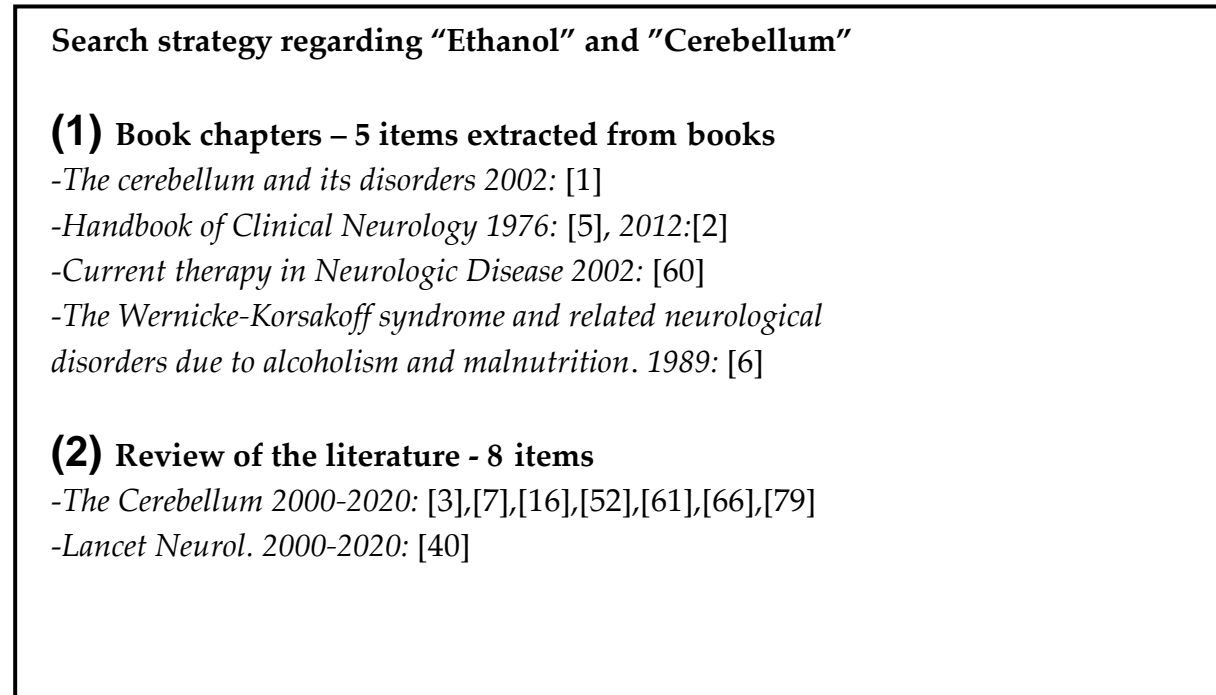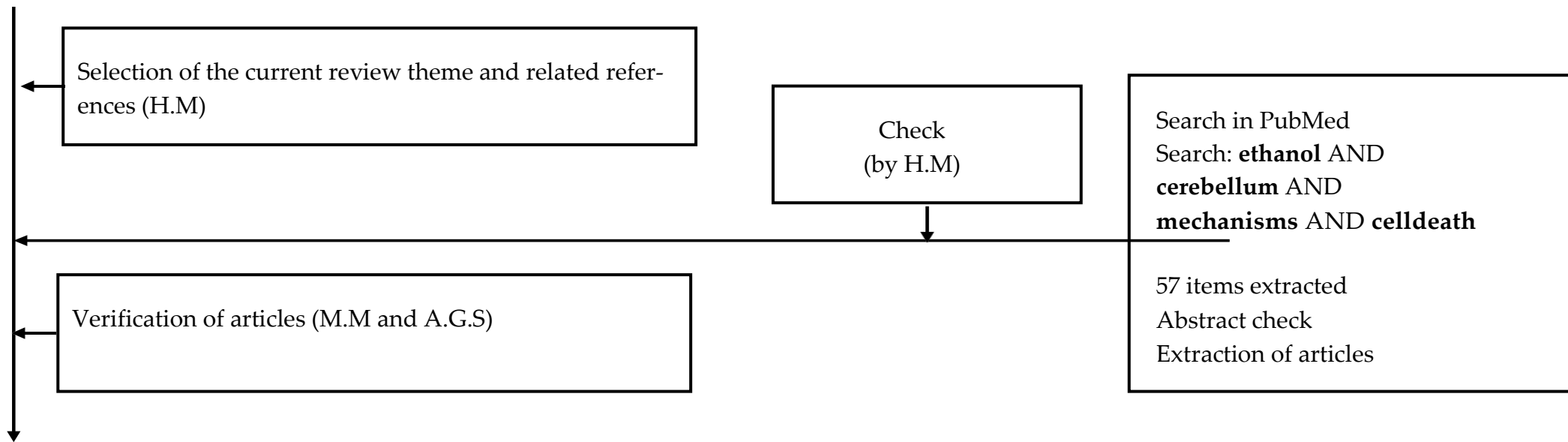

**Figure S1.** Review Algorithm

Supplement: Supplementary file 1 [file ijerph-18-08678-s001.zip › ijerph-1317525-supplementary.pdf]
